# Biodegradable Core–Multishell Nanocarriers: Influence of Inner Shell Structure on the Encapsulation Behavior of Dexamethasone and Tacrolimus

**DOI:** 10.3390/polym9080316

**Published:** 2017-07-29

**Authors:** Michael L. Unbehauen, Emanuel Fleige, Florian Paulus, Brigitta Schemmer, Stefan Mecking, Sam Dylan Moré, Rainer Haag

**Affiliations:** 1 Freie Universität Berlin, Institute for Chemistry and Biochemistry, Takustraße 3, 14195 Berlin, Germany; m.unbehauen@fu-berlin.de (M.L.U.); emafl@zedat.fu-berlin.de (E.F.); florian.paulus@fu-berlin.de (F.P.); 2 DendroPharm GmbH, Arnimallee 14, 14195 Berlin, Germany; sam.more@dendropharm.de; 3 Chemical Materials Science, Department of Chemistry, University of Konstanz, Universitätsstraße 10, 78467 Konstanz, Germany; brigitta.schemmer@uni.kn (B.S.); stefan.mecking@uni-konstanz.de (S.M.)

**Keywords:** biodegradable CMS nanocarrier, drug delivery, dendritic polymers, dexamethasone, tacrolimus

## Abstract

We here present the synthesis and characterization of a set of biodegradable core–multishell (CMS) nanocarriers. The CMS nanocarrier structure consists of hyperbranched polyglycerol (hPG) as core material, a hydrophobic (12, 15, 18, 19, and 36 C-atoms) inner and a polyethylene glycol monomethyl ether (mPEG) outer shell that were conjugated by ester bonds only to reduce the toxicity of metabolites. The loading capacities (LC) of the drugs, dexamethasone and tacrolimus, and the aggregate formation, phase transitions, and degradation kinetics were determined. The intermediate inner shell length (C15) system had the best overall performance with good LCs for both drugs as well as a promising degradation and release kinetics, which are of interest for dermal delivery.

## 1. Introduction

Cutaneous drug delivery is the method of choice when skin is the target and systemic side effects are to be avoided. This is especially the case in inflammatory skin diseases such as psoriasis and atopic dermatitis. The drugs, e.g., the commonly used dexamethasone and tacrolimus, must penetrate the skin first, which is the body’s natural barrier against xenobiotics. Especially the outermost layer, the stratum corneum, has to be overcome to reach the target, namely, the skin’s viable layers. Amphiphiles, among other penetration enhancers, are extensively used to facilitate a deeper penetration [1]. The application of nanoparticles, which have attracted much attention in recent years [2], has been widely explored. A nanoparticle can either consist of only the drug or be a carrier particle that contains the respective agents. The nanoparticles are classified according to their size, shape, and charge and are categorized into hard and soft, biological, organic, and inorganic particles. The skin offers different pathways for various types of nanoparticles that address one or more of the transdermal pathways, the intracellular, intercellular, or the follicular pathway, and also target the sebaceous gland [3]. Particulate formulation has been shown to enhance both the uptake of drugs into skin in general [4,5] but also specifically into hair follicles in a size-dependent manner [6,7]. Some of the polymeric particles that are reported in the literature contain esters or are ester-based (e.g., PCL [8], PLA [6], PLGA [7]) and thus are principally biodegradable. 

**Figure 1:**
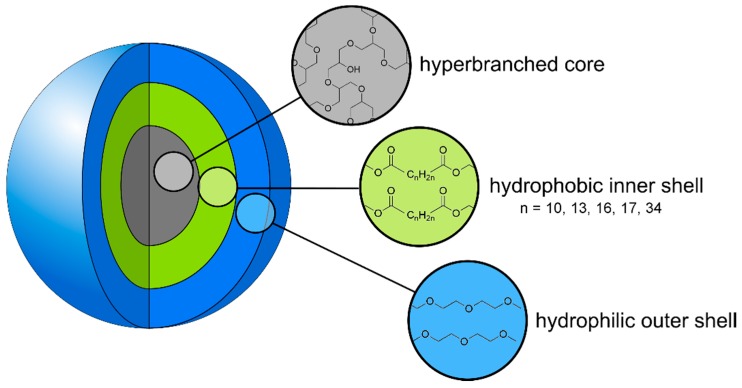
Structure of an ester-based CMS nanocarrier.

A more sophisticated structure is the so-called core–multishell (CMS) nanocarrier that extends the idea of the unimolecular micelle to the parent structure of a unimolecular liposome [9,10,11]. While a unimolecular micelle consists of simple, usually hydrophilic polymeric chains anchored to a dendritic core [12], the CMS nanocarrier is a unimolecular structure that has a branched core-unit with amphiphilic polymeric chains attached to it, which at least consist of one hydrophilic and one hydrophobic block (see Figure 1). Frequently used core structures are the polyester Boltorn^®^ H40 [13,14], poly (ethyleneimine) (PEI) and the highly hydrophilic hyperbranched polyglycerolamine (hPG-NH_2_) [15,16]. Initially designed as a unimolecular liposome, the CMS nanotransporter has been reported by our group for the transport of both hydrophilic and lipophilic guest molecules [11,15]. The CMS nanocarrier can solubilize hydrophobic guest molecules in a hydrophilic environment and vice versa [16]. In contrast to other drug delivery systems (DDS), the mode of solubilization is physical entrapment upon which larger aggregates are formed. The CMS nanocarrier and its differently functionalized derivatives have been successfully used for the transport of the fluorescence dye ITCC in a tumor xenograft model as well as the penetration enhancement of the model drug Nile Red into skin [16,17]. Their aggregation phenomena and host–guest interactions have been studied both experimentally and theoretically [18,19].

The long-term toxicity of the polymeric DDS is an important issue when it comes to in vivo applications. Not only the polymer assemblies but also their degradation products have to be taken into consideration. The usage of polyamines can be especially problematic: Their polycationic character enables interaction with the positively charged cellular membranes and can be a source of cytotoxicity [20]. In the case of hPG–NH_2_, another drawback is the synthetic effort. Three synthetic steps are necessary to convert the hyperbranched glycerol (hPG) to its amino-functionalized derivative [21].

Complementary to a previous biological study [22], here we present esterification as a promising alternative to amide formation for the synthesis of the CMS nanocarriers. Furthermore, the influence of different chain length, branching of inner shell, the type of chemical bond on melting point, and drug loading behavior of dexamethasone and tacrolimus are investigated.

## 2. Materials and Methods

All chemicals were used as bought without any further purification. Polyethylene glycol monomethyl ether (mPEG) 350 was bought from Sigma-Aldrich, Munich, Germany. 1,18-Octadecandioic acid was a kind gift from Cognis, Monheim am Rhein, Germany. 1,19-nonadecanedioic acid was received as the dimethyl ester from the Mecking group, Konstanz, Germany [23]. The branched C18b-diacid was a gift from Cognis (see Scheme S1 for composition and purification). 1,12-Dodecandioic acid was bought from Sigma Aldrich, St. Louis, MI, USA and 1,15-pentadecandioic acid was bought from ToniChemPharma (Huizhou, China). hPG and CMS-A18 were produced by previously published methods [21,24]. 

Methanol was bought from Sigma-Aldrich; dry pyridine was bought from Acros, Geel, Belgium and stored over calcium hydride (Acros). Dry DCM was taken from solvent purification system (SPS-800) by MBRAUN (Stratham, NH, USA) and stored over a molecular sieve (4 Å, Roth). 

### 2.1. Nuclear Magnetic Resonance (NMR)

NMR spectra were recorded either on a Jeol Eclipse 500 MHz (Tokyo, Japan) or a Bruker AVANCE III 700 MHz spectrometer (Billerica, MA, USA). Proton and carbon NMR were recorded in ppm and were referenced to the indicated solvents [25]. NMR data were reported including: chemical shift, multiplicity (s = singlet, d = doublet, t = triplet, m = multiplet), integration, and coupling constants (s) in Hertz (Hz). Multiplets (m) were reported over the range (ppm) in which they appear in the spectrum. All spectra were recorded at 300 K.

### 2.2. Infrared (IR) Spectroscopy

IR spectra were recorded on a Jasco FT/IR 4100LE spectrometer (Groß-Umstadt, Germany) equipped with a MIRacleTM single reflection ATR device from PIKE Technologies (Fitchburg, WI, USA). Samples were directly placed on the ATR crystal. Data recording and analysis was done with Spectra Manager^®^ II software from Jasco.

### 2.3. Dynamic Light Scattering (DLS)

For the determination of hydrodynamic sizes, dynamic light scattering (DLS) measurements were performed on a Malvern Zetasizer Nano (Herrenberg, Germany) equipped with a laser at 532 nm using backscattering mode (detector angle 173°). The samples were filtered through 0.45 μm regenerated cellulose syringe filters prior to DLS measurement and 100 μL of the resulting solution added to a disposable micro-cuvette. Autocorrelation functions were analyzed using Zetasizer DTS software (Malvern, Herrenberg, Germany) to determine the size distribution by intensity or number. The fraction (%) indicates the proportion of measured size relative to the total signal scattered by the CMS nanocarriers. Measurements were performed at 25 °C if not stated otherwise.

### 2.4. Gel Permeation Chromatography

A Shimadzu (Kyoto, Japan) liquid chromatography (LC) system was employed for the gel permeation chromatography (GPC) measurements. Three PolarSil columns (PSS Polymer Standards Service GmbH, Germany; PolarSil 8 × 300 mm, 100 Å, 1000 Å, 3000 Å with 5 μm particle size) and a refractive index detector (RI) were used to separate and analyze polymer samples. As the mobile phase DMF (0.3 wt % LiBr and 0.6 wt % acetic acid) was used at a flow rate of 1 mL·min^−1^. Columns and RI detector were heated to 40 °C. The system was calibrated against polystyrene calibration standards (PSS, Germany). Samples were measured at a concentration of 10 mg·mL^−1^. LC solution software from Shimadzu was used for data analysis.

### 2.5. Film Encapsulation Method

The film uptake method was chosen for the encapsulation procedure. To form a film, 50 wt % of the guest compound (dexamethasone or tacrolimus) was dissolved in a vial and the solvent removed on a rotavap. A stock solution of the respective CMS nanocarrier (5 mg·mL^−1^) was filled onto the film and the suspension stirred for 22 h at room temperature. Excess guest was removed via filtration through a 450 nm RC syringe filter. The same procedure was carried out at 60 °C for the CMS-E19 nanocarrier, which was poorly soluble at low temperatures.

### 2.6. HPLC Analysis

The guest concentration was determined via high performance liquid chromatography (HPLC, Phenomenex Gemini, Torrance, CA, USA, C18, 5 µm 110 Å, 250 mm × 6.4 mm, flow 1 mL·min^−1^, 210 nm, 40% acetonitrile/H_2_O for dexamethasone, acetonitrile for tacrolimus) using a UV detector set to λ = 210 nm. For the preparation of the sample, the samples were diluted with acetonitrile (dexamethasone) or lyophilized and redissolved in acetonitrile (tacrolimus). 

### 2.7. Determination of Enzymatic Activity

The enzymatic activity of *Rhizomucor miehei* lipase was determined photometrically similar to a previously published method [26]. First, 5–10 μL of the diluted enzyme solution was added to a solution of 1 mM 4-nitrophenyl acetate in PBS (1% acetonitrile, total volume 1 mL). After 1 h stirring at 200 RPM and 32 °C, the concentration of the reaction product 4-nitrophenol was determined photometrically using a spectrophotometer and the extinction coefficient of 4-nitrophenol (ε = 11.9 × 10^3^ M^−1^·cm^−1^, λ = 400 nm). A sample without enzyme was used to deduct the fraction of thermal hydrolysis. One unit of enzymatic activity equals the release of 1 μmol 4-nitrophenol per minute.

### 2.8. Enzymatic Degradation

To determine the rate of enzymatic degradation, a solution of CMS nanocarrier (5 mg·mL^−1^) in PBS was prepared and lipase from Rhizomucor miehei (8 mU·mg^−1^ polymer) was added. The solutions were stirred with a small stir bar at 200 RPM at 32 °C. Samples were taken at different time points, lyophilized, redissolved in DMSO-d_6_ and analyzed via NMR. Due to the high consumption of material, the experiment was only performed once.

### 2.9. Release by Enzymatic Degradation

To measure the release of the drug from an enzymatically degraded carrier, a solution of dexamethasone-loaded CMS nanocarrier in PBS was prepared (10 mg mL^−1^ polymer), Rhizomucor miehei lipase (8 mU·mg^−1^ polymer) added (or not added in the case if the untreated control (UC)) and the solution stirred at 200 RPM. At different time points, the solution was centrifuged for 10 min at 4000 RPM (Heraeus Biofuge Primo), the supernatant sampled and stirring was continued. Samples were either diluted with acetonitrile before measurement with HPLC to match the eluent.

### 2.10. Synthesis

#### 1,19-Nonadecandioic Acid

Following a previously published procedure [23], dimethyl-1,19-nonadecanedioate (854.9 mg, 2.4 mmol) was suspended in 6.4 mL methanol and the suspension heated to 70 °C (oil bath). After dissolution, a solution of 1.8 g (31.4 mmol) KOH in 6.4 mL Methanol was added dropwise and the solution was stirred overnight at 70 °C. The methanol was distilled off and the residue dissolved in water. Then it was acidified via the addition of 3 M HCl to a pH of 1. The precipitate was filtered off with a glass frit, washed with water, and dried in the air stream of the pump. The product could be isolated as 764 mg (2.3 mmol) of a colorless solid (yield 97%).

^1^H NMR: (DMSO-d_6_, 500 MHz, TMS): δ 2.17 (t, 2H, *J* = 7.4 Hz, 4H, HOOC–C*H_2_*–); 1.51–1.43 (m, 4H, HOOC–CH_2_–C*H_2_*–); 1.28–1.20 (br s, 26H, –CH_2_– backbone). ^13^C NMR: (DMSO-d_6_, 125 MHz, TMS): δ 174.42 (HOO*C*–CH_2_–), 33.66 (HOOC–*C*H_2_–), 29.03–28.54 (HOOC–CH_2_–*C*H_2_–), 24.49 (–CH_2_– backbone).

### 2.11. Double-Shell Building Blocks

#### 2.11.1. C12-mPEG350

In a three-neck flask equipped with a gas inlet, thermometer with quick fit, and septum, a mixture of mPEG350 (0.3 mol, 1 equiv.) with dodecanedioic acid (C12, 0.9 mol, 3 equiv.) was stirred under high vacuum and heated to 120 °C. The mixture was kept at this temperature for at least 1.5 h until a clear melt was obtained. The temperature was then raised to 180 °C and stirred for an additional 4.5 h. The reaction mixture was kept under vacuum and allowed to cool to 120 °C. While still a melt, the hot reaction mixture was transferred to a beaker and cooled to room temperature. The still warm waxy solid was chopped, 2 L of methylene chloride added, and the resulting mixture vigorously stirred until a near homogeneous suspension was formed. The suspension was filtered and the filtrate concentrated to a final volume of 600 mL by rotary evaporation. The solution was kept a 5 °C for 18 h. Precipitated excess C15 was removed by filtration. The remaining filtrate was concentrated by rotary evaporation and subsequently dried under high vacuum to yield a colorless wax (69%) of C12-mPEG350. 

^1^H NMR (500 MHz, methanol-d_4_, TMS): δ (ppm) = 4.22–4.18 (m, 2H, –CH_2_–OCO–), 3.75–3.50 (m, 30H, mPEG backbone), 3.36 (s, 3H, –O–CH_3_), 2.33 (t, 2H, *J* = 7.4 Hz, ROOC–C*H_2_*–CH_2_–), 2.28 (t, 2H, *J* = 7.4 Hz, HOOC–C*H_2_*–CH_2_–), 1.65–1.55 (m, 4H, –CO–CH_2_–C*H_2_*–), 1.37–1.20 (m, 12H, –CH_2_–(C*H_2_*)_6_–CH_2_–). ^13^C NMR (125 MHz, methanol-d_4_, TMS): δ (ppm) = 177.4, 175.2, 72.9, 71.7–71.3, 70.3, 70.1, 64.5, 64.3, 59.1, 59.1, 34.9, 34.9, 30.7, −30.1, 26.0, 26.0. IR (cm^−1^): 2923, 2856, 1732, 1456, 1349, 1247, 1099, 1040, 946, 849, 724. GPC: *M*_n_ = 680 g·mol^−1^, *M*_w_ = 750 g·mol^−1^, *PDI* = 1.10.

#### 2.11.2. C15-mPEG350

C15-mPEG350 was synthesized in analogy to C12-mPEG350. The reaction of mPEG350 (0.3 mol, 1 equiv.) and 1,15-pentadecanedioic acid (0.9 mol, 3 equiv.) resulted in C15-mPEG350 as a pale-yellow wax (68% yield).

^1^H NMR (500 MHz, methanol-d_4_, TMS): δ (ppm) = 4.22–4.18 (m, 2H, –CH_2_–OCO–), 3.75–3.50 (m, 30H, mPEG backbone), 3.36 (s, 3H, –O–CH_3_), 2.33 (t, 2H, *J* = 7.4 Hz, ROOC–C*H_2_*–CH_2_–), 2.28 (t, 2H, *J* = 7.4 Hz, HOOC–C*H_2_*–CH_2_–), 1.65–1.55 (m, 4H, –CO–CH_2_–C*H_2_*–), 1.37–1.20 (m, 18H, –CH_2_–(C*H_2_*)_9_–CH_2_–). ^13^C NMR (125 MHz, methanol-d_4_, TMS): δ (ppm) = 177.5, 175.3, 72.9, 71.6–71.5, 71.3, 70.1, 64.5, 59.1, 34.9, 34.9, 30.7, −30.1, 26.0, 26.0. IR (cm^−1^): 2922, 2854, 1733, 1456, 1349, 1248, 1099, 1040, 946, 850, 723. GPC: *M*_n_ = 680 g·mol^−1^, *M*_w_ = 735 g·mol^−1^, *PDI* = 1.08.

#### 2.11.3. C18-mPEG350

Similar to an already published method [15], 14.9 g (42.7 mmol) methoxy-poly(ethylene glycol) (mPEG) 350 (dried overnight at 70 °C under low pressure (5 × 10^−2^ mbar) and 47.7 g (15.2 mmol) 1,18-octadecanedioic acid were added without solvent into a Schlenk flask. The reaction mixture was heated up to 185 °C and the reaction mixture was stirred vigorously for 3 h under vacuum (5 × 10^−2^ mbar). After 3 h, a sample was taken and submitted for NMR, the mixture was allowed to cool down to 140 °C, and a reflux condenser was installed. Then, 300 mL toluene was added into the flask. While still stirring, the reaction mixture was slowly allowed to cool down to 0 °C. The resulting suspension was filtrated and the white residue was washed with 300 mL of cold (0 °C) toluene. The filtrate and washings were combined and concentrated by rotary evaporation in vacuo and the remaining solvent removed in vacuo at 50 °C. Of the pre-purified product (19.8 g, 72% yield), 5 g were purified via HPLC (Phenomenex Gemini, C18, 5 μm, 110A, 250 mm × 21.2 mm, flow 20 mL min^−1^, 210 nm, 95% MeOH/H_2_O) to yield 3.9 g of a white wax (yield 56%).

^1^H NMR (700 MHz, methanol-d_4_, TMS): δ (ppm) = 4.21 (m, 2H, –CH_2_–OOC–), 3.71–3.52 (m, 28.4H, mPEG backbone), 3.36 (s, 3H, –O–CH_3_), 2.34 (t, 2H, *J* = 7.4 Hz, 2H, R–OOC–C*H_2_*–CH_2_–), 2.28 (t, 2H, *J* = 7.4 Hz, 2H, –HOOC–C*H_2_*–CH_2_–), 1.65–1.54 (m, 4H, –OOC–CH_2_–C*H_2_*–), 1.38–1.25 (br s, 24H, –CH_2_–(C*H_2_*)_12_–CH_2_–). ^13^C NMR (176 MHz, methanol-d_4_, TMS): δ (ppm) = 177.57, 175.35, 72.96, 71.75–71.25, 70.15, 64.55, 35.00–34.90, 30.80–30.15, 26.12–26.00. IR (cm^−1^): 2916, 2848, 1730, 1702, 1462, 1342, 1243, 1106, 954, 848, 729. GPC: *M*_n_ = 830 g·mol^−1^, *M*_w_ = 920 g·mol^−1^, *PDI* = 1.11.

#### 2.11.4. C19-mPEG350

C19-mPEG350 was synthesized analog to C18-mPEG350. In total, 1.8 g (5.0 mmol) mPEG350 and 4.9 g (15.0 mmol) 1,19-nonadecanedioic acid led to 2.4 g (3.7 mmol) of the colorless solid product (74% yield).

^1^H NMR (700 MHz, methanol-d_4_, TMS): δ 4.21 (m, 2H, –CH_2_–OOC–), 3.71–3.52 (m, 26.8H, mPEG backbone), 3.36 (s, 3H, –O–CH_3_), 2.33 (t, 2H, *J* = 7.4 Hz, 2H, R–OOC–C*H_2_*–CH_2_–), 2.28 (t, 2H, *J* = 7.4 Hz, 2H, –HOOC–C*H_2_*–CH_2_–), 1.64–1.57 (m, 4H, –OOC–CH_2_–C*H_2_*–), 1–1.27 (br m, 26H, –CH_2_–(C*H_2_*)_12_–CH_2_–). ^13^C NMR: (176 MHz, methanol-d_4_, TMS): δ 177.58, 175.34, 72.97, 71.65–71.50, 71.37, 70.16, 64.56, 59.10, 34.99, 34.94, 30.85–30.15, 26.10, 26.03. IR (cm^−1^): 2917, 2848, 1729, 1694, 1463, 1345, 1234, 1105, 961, 847, 730. GPC: *M*_n_ = 840 g·mol^−1^, *M*_w_ = 920 g·mol^−1^, *PDI* = 1.10.

#### 2.11.5. C18b-mPEG750

8.5 g C18b-diacid (15.0 mmol) and 2.8 g mPEG750 (3.7 mmol) were added into a Schlenk flask and stirred at 60 °C for 30 min. Afterwards, the reaction mixture was heated to 180 °C and stirred under high vacuum (<0.5 × 10^−2^ mbar) for 3 h. After cooling down, the mixture was purified via column chromatography using the eluents chloroform/acetic acid (80:1), chloroform/methanol 20:1, and methanol, which yielded the product as 3.8 g of a yellow oil (yield 78%).

^1^H NMR: (700 MHz, methanol-d_4_, TMS): δ 6.85 (m, 0.4H, arom. –H), 4.21 (m, 2H, –CH_2_–OOC–), 3.75–3.50 (m, 63.2H, mPEG backbone), 3.36 (s, 3H, –O–CH_3_), 2.54 (m, 1.6H, benzyl–H), 2.33 (t, 2H, *J* = 7.1 Hz, 2H, R–OOC–C*H_2_*–CH_2_–), 2.28 (t, 2H, *J* = 7.1 Hz, 2H, HOOC–C*H_2_*–CH_2_–), 1.64–1.50 (m, 8.2H, –OOC–CH_2_–C*H_2_*– + –CH_2_–C*H_2_*–Ph), 1.5–1.1 (br s, 44.5H, aliph. backbone), 0.91 (m, 3H, –CH_2_–C*H_3_*). GPC: *M*_n_ = 1380 g mol^−1^, *M*_w_ = 1610 g mol^−1^, *PDI* = 1.17. ^13^C NMR: (176 MHz, methanol-d_4_, TMS): δ 177.50, 175.28, 72.98, 71.87–71.05, 70.17, 64.57, 59.11, 34.98, 33.08, 31.05–29.95, 26.12, 23.76, 14.52. IR (cm^−1^): 2921, 2855, 1732, 1456, 1349, 1249, 1101, 947, 850, 722.

### 2.12. Nanocarriers

#### 2.12.1. CMS-E12

To link the shell components with the hPG core (*M*_n_ = 9.9 kDa; *M*_w_ = 15.8 kDa), 141 g C12-mPEG350 (0.3 mol, 1 equiv.) were dissolved in 625 mL anhydrous methylene chloride in a three-necked flask equipped with a gas inlet and two septa. The solution was cooled down to 0 °C and 25.9 mL thionyl chloride (42.5 g, 0.4 mol, 1.5 equiv.) was added. One septum was then replaced with a reflux condenser connected to two washing flasks, the second of which was filled with sodium hydroxide solution. The reaction was refluxed for 4.5 h. Methylene chloride and the excess of thionyl chloride were removed by cryo-distillation, and the residue dried for a further 3 h under high vacuum. In the meantime, 26 g hPG (2.6 mmol) were dissolved in 530 mL dry pyridine. The resulting acid chloride was dissolved in 200 mL dry methylene chloride and added dropwise to the hPG solution. The reaction was stirred overnight and then quenched by the addition of methanol (1.48 mol). Solvents were then removed with a rotary evaporator. The crude product was purified with a Millipore bench scale tangential flow field filtration system, equipped with a 30 kDa·MW cut-off membrane and at least 250 mL of distilled water per gram CMS nanocarrier. The product was freeze-dried, which yielded 117 g of a clear viscous yellow oil of CMS-E12 (62%). 

^1^H NMR (500 MHz, DMSO-d_6_, TMS): δ (ppm) = 4.09 (s, 2H, –CH_2_–OCO–), 3.69–3.25 (m, 37H, mPEG repeating unit and hPG backbone), 3.23 (s, 3H, –O–CH_3_), 2.25 (m, 4H, –CH_2_–COO–), 1.49 (m, 4H, –C*H_2_*–CH_2_–COO–), 1.22 (m, 12H, –(CH_2_)_6_–). ^13^C NMR (125 MHz, DMSO-d_6_, TMS): δ (ppm) = 172.7, 71.3, 69.9–69.6, 68.4, 63.0, 58.0, 33.4, 29.3–28.4, 24.5. IR (cm^−1^): 3462, 2922, 2856, 1732, 1456, 1349, 1248, 1100, 948, 850, 723. GPC: *M*_n_ = 32,200 g·mol^−1^, *M*_w_ = 42,200 g·mol^−1^, *PDI* = 1.31.

#### 2.12.2. CMS-E15

CMS-E15 was synthesized in analogy to CMS-E12. In total, 151 g (0.3 mol) C15-mPEG350 were reacted with 25.9 mL thionyl chloride (42.5 g, 0.4 mmol) and 26 g (2.6 mmol) hPG (*M*_n_ = 10.4 kDa; *M*_w_ = 16 kDa) to yield 138 g of a yellow to brown oil (79%). 

^1^H NMR (500 MHz, DMSO-d_6_, TMS): δ (ppm) = 4.09 (s, 2H, –CH_2_–OCO–), 3.69–3.25 (m, 30H, mPEG repeating unit and hPG backbone), 3.23 (s, 3H, –O–CH_3_), 2.25 (m, 4H, –CH_2_–COO–), 1.49 (m, 4H, –C*H_2_*–CH_2_–COO–), 1.21 (m, 18H, –(CH_2_)_9_–). ^13^C NMR (125 MHz, DMSO-d_6_, TMS): δ (ppm) = 172.7, 71.3, 69.9–69.6, 68.3, 63.0, 58.0, 33.4, 29.3–28.4, 24.5. IR (cm^−1^): 3460, 2922, 2853, 1733, 1456, 1349, 1248, 1100, 949, 851, 722. GPC: *M*_n_ = 41,100 g·mol^−1^, *M*_w_ = 59,100 g·mol^−1^, *PDI* = 1.44.

#### 2.12.3. CMS-E18

hPG (480 mg, 6.5 mmol OH groups, *M*_n_ = 8.1 kDa; *M*_w_ = 16.2 kDa) was dried by dissolving in 5 mL of anhydrous pyridine and evaporating the solvent. Then it was dissolved in 38 mL dry pyridine. C18-mPEG350 (1.7 mmol, 7.5 g, previously dried at 50 °C under vacuum <5 × 10^−2^ mbar overnight) in a three-neck flask (equipped with a reflux-condenser, an olive and a septum) was dissolved in 38 mL dry methylene chloride. At 0 °C, 1.27 mL thionyl chloride were added and after 10 min the temperature was increased to the boiling point of methylene chloride. After 2 h, the solvent was evaporated by cryo-distillation. At 0 °C, the intermediate was dissolved in dry pyridine and the solution was added dropwise to the solution of hPG. After stirring overnight, the solvent was distilled off and the crude product purified with dialysis (5 kDa, 3 × 3 L) and ultrafiltration (MWCO 30 kDa, methanol) yielding the product as a viscous, brownish oil (2.7 g, 78%). 

^1^H NMR (700 MHz, methanol-d_4_, TMS): δ (ppm) = 4.21 (s, 2H, –CH_2_–OCO–), 3.72–3.52 (m, 31.7H, mPEG repeating unit and hPG backbone), 3.36 (s, 3H, –O–CH_3_), 2.34 (m, 4H, –OOC–CH_2_–), 1.62 (m, 4H, –OOC–CH_2_–C*H_2_*–), 1.31 (m, 24H, –(CH_2_)_12_–). ^13^C NMR (176 MHz, methanol-d_4_, TMS): δ (ppm) = 175.0, 73.0, 71.6–71.4, 70.2, 64.6, 59.2, 35.0, 31.0–30.4, 26.2. IR (cm^−1^): 3445, 2922, 2852, 1733, 1456, 1349, 1248, 1103, 949, 851, 722. GPC: *M*_n_ = 34,900 g·mol^−1^, *M*_w_ = 49,200 g·mol^−1^, *PDI* = 1.41.

#### 2.12.4. CMS-E19

88.3 mg hPG (1.2 mmol OH groups, *M*_n_ = 6.9 kDa; *M*_w_ = 12.4 kDa) was dried by dissolving in 1 mL of anhydrous pyridine and evaporating the solvent. Then the residue was dissolved in 7 mL dry pyridine. C19-mPEG350 (2.4 mmol, 1.6 g, previously dried at 50 °C under vacuum <5 × 10^−2^ mbar overnight) in a three-neck flask (equipped with a reflux-condenser, an olive, and a septum) was dissolved in 8 mL dry methylene chloride. At 0 °C, 0.3 mL thionyl chloride were added and after 10 min the temperature increased to the boiling point of methylene chloride. After 2 h, the solvent was evaporated by cryo-distillation. At 0 °C, the intermediate was dissolved in dry methylene chloride and the solution was added dropwise to the solution of hPG. After stirring overnight, the solvent was distilled off and the crude product purified with dialysis (2 kDa, 3 × 3 L) and ultrafiltration (MWCO 30 kDa, methanol) yielding the product as a colorless solid (284 mg, 64%). 

^1^H NMR (700 MHz, methanol-d_4_, TMS): δ (ppm) = 4.20 (s, 2H, –CH_2_–OCO–), 3.72–3.51 (m, 31.3H, mPEG repeating unit and hPG backbone), 3.35 (s, 3H, –O–CH_3_), 2.33 (m, 4H, –OOC–CH_2_–), 1.62 (m, 4H, –OOC–CH_2_–C*H_2_*–), 1.32 (m, 24H, –(CH_2_)_13_–). ^13^C NMR (176 MHz, methanol-d_4_, TMS): δ (ppm) = 174.9, 73.1z, 71.8–71.4, 70.3, 64.6, 59.3, 35.1, 31.7–30.4, 26.2. IR (cm^−1^): 3399, 2917, 2850, 1712, 1486, 1306, 1226, 1111, 953, 851, 746. GPC: *M*_n_ = 32,300 g·mol^−1^, *M*_w_ = 42,600 g·mol^−1^, *PDI* = 1.32.

#### 2.12.5. CMS-E18b

CMS-E18b was synthesized analog to CMS-E19. In total, 2.2 mg (1.7 mmol) C18b-mPEG750 were reacted with 186 μL thionyl chloride (305 mg, 2.6 mmol) and 105.6 g (1.4 mmol OH groups, *M*_n_ = 6.9 kDa; *M*_w_ = 12.4 kDa) hPG to yield after purification with UF (methanol, 30 kDa MWCO) 577 mg of a brown oil (42% yield).

^1^H NMR (500 MHz, methanol-d_4_, TMS): δ (ppm) = 7.08–6.69 (m, 0.4H, arom.-H); 4.21 (s, 2H, –CH_2_–OCO–), 3.72–3.50 (m, 70.4H, mPEG repeating unit and hPG backbone), 3.36 (s, 3H, –O–CH_3_), 2.55 (m, 1.6H, benzyl–H); 2.33 (m, 4H, –OOC–CH_2_–), 1.61 (m, 4H, –OOC–CH_2_–C*H_2_*–, –CH_2_–C*H_2_*–Ph), 1.31 (br s, 44.5H, aliph. backbone); 0.91 (m, 3H, –CH_2_–C*H_3_*). ^13^C NMR (125 MHz, methanol-d_4_, TMS): δ (ppm) = 174.4, 73.0, 71.7–71.3, 70.2, 64.6, 59.2, 35.1, 31.2–30.2, 26.2, 23.9, 15.0. IR (cm^−1^): 3454, 2922, 2855, 1732, 1456, 1349, 1248, 1098, 949, 849, 756. GPC: *M*_n_ = 33,300 g·mol^−1^, *M*_w_ = 56,100 g·mol^−1^, *PDI* = 1.68.

#### 2.12.6. CMS-A18

Amide-based CMS nanocarriers CMS-A18 were synthesized according to procedures published elsewhere [16]. 

## 3. Results

### 3.1. Synthesis of the Ester-Based Core–Multishell Nanocarriers

#### 3.1.1. Synthesis of the Shell Molecule

**Scheme 1:**
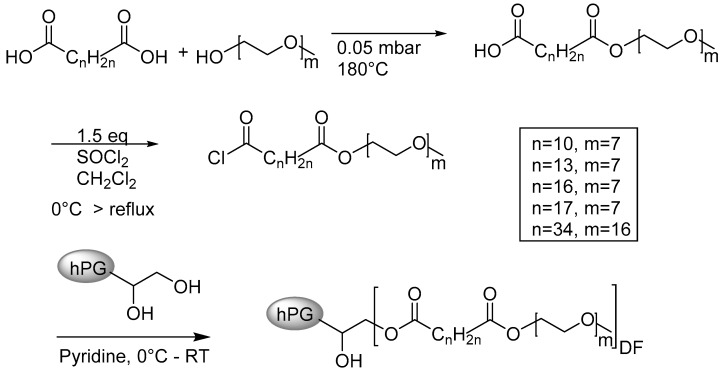
Synthetic route of all ester-based CMS nanocarriers.

The double shells of the various CMS architectures were synthesized according to a previously published procedure [15]. The esterification reaction was conducted without a catalyst at 180 °C and under low pressure (<10^−1^ mbar, see Scheme 1). To avoid diester formation, stoichiometries of 3–4:1 (diacid to mPEG) were chosen. Purification was either carried out by precipitation (C12 and C15) or chromatography. Normal column chromatography was sufficient for the C18b-mPEG750 double shell but reverse-phase HPLC had to be applied for C18-mPEG350 and C19-mPEG350 to remove residual diacid.

#### 3.1.2. Synthesis of the CMS Architectures

Using hPG as a core molecule instead of its amino derivative not only avoids the potentially toxic hPG–NH_2_ as a degradation product, it also supersedes its synthesis from hPG amine and thus three synthetic steps [21].

The alcohol of bare hPG, however, features a lowered nucleophilicity in comparison to its aminated counterpart. Therefore, a higher reactivity of the carboxylic group becomes necessary. Activation to the acid chloride prior to the reaction with thionyl chloride led to the desired product (see Scheme 1). The amounts of 0.8–2 eq. acid chloride-activated shell (depending on batch size) were used because of its sensitivity towards water. Ultrafiltration compared to dialysis yielded purer compounds and the molecular-weight cut-offs were between 3 kDa and 30 kDa in methanol, depending on the molecular weight of the double-shell. 

### 3.2. Degree of Functionalisation

In order to estimate the degree of functionalization, the molecular weights of all CMS were determined by gel permeation chromatography (GPC) in DMF. All systems were as well analyzed with NMR spectroscopy. The results are given in Table 1, the estimated error of the determination of the DF by NMR is in the range of 10% (see Figure S2).

The molecular weights determined by GPC analysis were all in the same range. The degree of functionalization (DF) was determined from 1H NMR by comparing the combined hPG/mPEG peak to the signal of the aliphatic protons of the inner shell. The residual signal originated from core protons and therefore the DF could be calculated using Equation (1) for CMS nanocarrier with an inner shell length of 12 to 19 carbon atoms and Equation (2) for CMS-E18b.

(1) $$DF=\frac{5}{\sigma_{a,e,f,g}-31.8}$$

(2) $$DF=\frac{5}{\sigma_{g}-63.2}$$

The *M*_n_ of CMS nanocarriers and hPG were used for calculating the DF values from the GPC data, and are all stated in Table S1. The GPC data indicates that all nanocarriers were defined (*PDI* < 2) and of comparable molecular weights (*M*_n_ ~ 40 kDa). The GPC-based DF values were smaller than the ones calculated from NMR spectra. This was due to the comparison to a linear standard (polystyrene) in the GPC analysis that made the nanocarriers appear smaller, which is why the estimation via NMR is more realistic.

### 3.3. DSC Measurements

**Figure 2:**
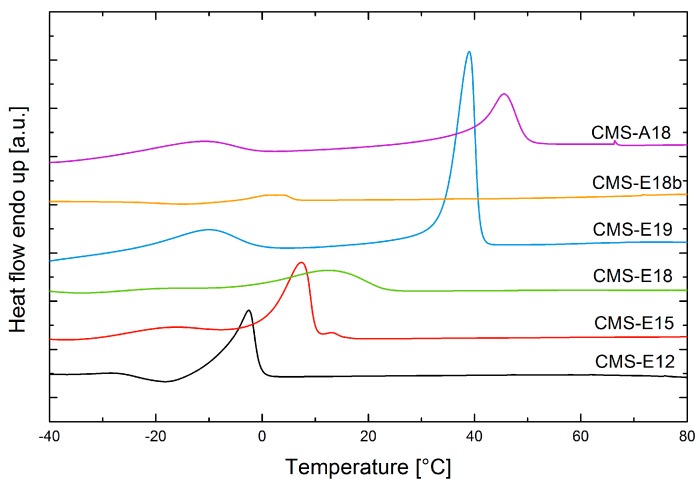
DSC measurements of CMS nanocarriers. Samples have been heated to 85 °C to erase any thermal history. Graphs show second heating at 5 °C min^−1^.

Differential scanning calorimetry was used to analyze the carriers regarding their phase-separation and thermal transitions (Figure 2). All carriers except CMS-E18b and CMS-E12 exhibited two peaks. Each of those peaks represents a segregated phase. The calorigrams of CMS-E18b and CMS-E12 only showed one peak. Possible reasons for this include that no phase segregation took place in these carriers or that the melting point (*T*_m_) of the aliphatic phase was too close to the *T*_m_ of the mPEG phase to result in separated peaks. The mPEG phase exhibited a *T*_m_ in the −20 to −10 °C region for all carriers, which was in accordance with the melting point of mPEG350 (−8 °C) [27]. The second peak represents the hydrophobic domain and proves phase-segregation. The temperature at which this transition occurred reflects the interaction between chains in the phase that needs to be overcome for a less ordered state. Comparing CMS-E12, CMS-E15, CMS-E18, and CMS-E19, this *T*_m_ increases as expected with higher chain lengths. Surprisingly, there is a significant step between CMS-E18 and CMS-E19 of 26.8 °C with a difference of an inner chain length of only one methylene group. This unexpectedly high *T*_m_ also influences other properties of the unimolecular micelle. While all other CMS nanocarriers are highly soluble in water, the solubility of CMS-E19 at RT is only 60 mg·L^−1^. At temperatures around 60 °C, it exceeds 5 mg mL^−1^, which is why the loading experiments were also carried out at this temperature.

### 3.4. DLS Analysis of Loaded and Unloaded CMS Nanocarriers

DLS analysis was applied to estimate hydrodynamic diameters in polar solvents and aggregation phenomena upon loading in water. The two pharmacophores dexamethasone and tacrolimus were chosen for two reasons: First, they are both commonly used in the therapy of inflammatory skin diseases. Secondly, they are very different on a molecular level. While dexamethasone is a small molecule and exhibits a rigid structure, tacrolimus features a relatively high molecular weight and dynamic conformal changes. Table S2 summarizes the number-averaged results in methanol, which reflect well the sizes of the unimolecular micelles. In water, the nanocarriers readily aggregated into smaller and bigger aggregates, which was not the case for methanol. The hydrodynamic diameters of ester-based nanocarriers were all in the range of 5 to 10 nm, which was expected for unimolecular micelles and classifies them as nano-scale objects.

As the intensity-based distribution of a DLS measurement focuses more on larger particles in suspension, the abundance of larger aggregates becomes visible. At the concentration of 5 mg mL^−1^, DLS data (Table 2) indicated the formation of small aggregates even without a guest molecule. This was in accordance to the findings published by Rabe et al. that suggested that CMS-A18 formed aggregates of around eight unimers [28]. The next generation ester-based nanocarriers showed comparable sizes that suggest a similar trend towards aggregates at higher concentrations.

When loaded with a hydrophobic guest molecule, the tendency towards aggregation increased. The smaller aggregates disappeared and the larger colloidal structures became more predominant in the DLS spectra, which is in accordance with previously published data [15,18]. These results suggest bridging of two or more unimolecular micelles by a hydrophobic drug molecule, similar to the formation of hydrophobic patches that drove aggregation. Tacrolimus-loaded CMS-A18 was the only exception to this trend, but it also featured one of the lowest loading capacities. The ζ-potential of all carriers were approximately neutral. Thus, electrostatic interactions are assumed to play a minor role in the aggregation phenomena.

### 3.5. Loading Capacities of Dexamethasone and Tacrolimus

The loading capacity (*LC*) of a carrier depends on the method and on the guest molecule encapsulated in it. It is defined as the solubilized mass concentration of a guest compared to the mass concentration of its host (see Equation (3)).

(3) $$LC=\frac{w\left(guest\right)-w_{0}\left(guest\right)}{w\left(carrier\right)}$$

**Figure 3:**
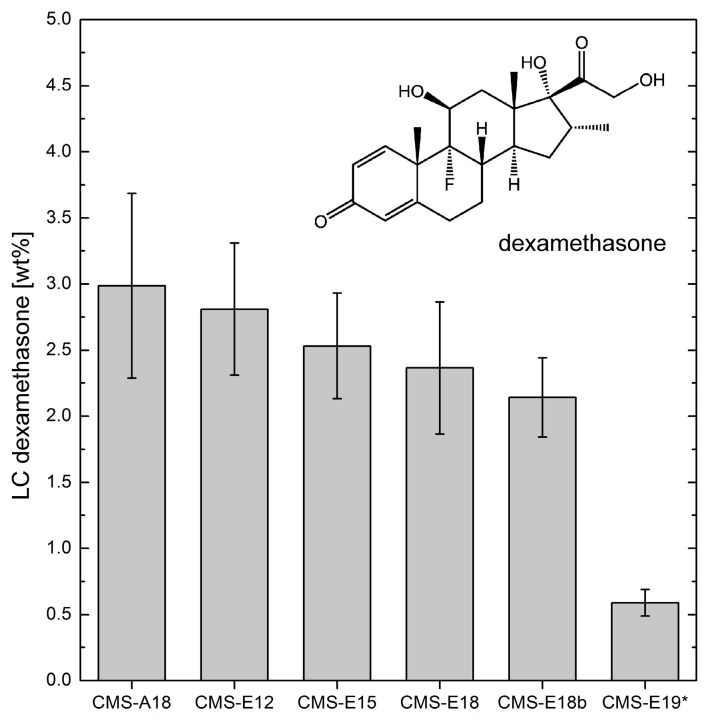
Loading capacities of the investigated carrier systems. Encapsulation was performed with the film uptake method, *n* = 3, * performed at 60 °C.

To determine *LC*, dexamethasone and tacrolimus were encapsulated via the film uptake method, respectively. Fifty weight percent of the drug was dissolved in ethanol and the solvent was removed to form a film. Then, the stock solution of the carrier in water was added and the suspension stirred for 22 h. After removal of the non-solubilized drug by filtration, the guest concentration was measured by HPLC and the loading capacities calculated (see Equation (3)). Dexamethasone, a rather small hydrophobic drug, can be encapsulated in all carriers (except for CMS-E19) with reasonable loading capacities (Figure 3 and Table S4 for numerical values). Comparing CMS-E12, CMS-E15, CMS-E18, and CMS-E19, one could find no clear trend between the chain length and the melting points obtained from DSC measurements (see Figure S3). The higher the hydrophobic interactions between the carriers, the lower were the interactions between drug and carrier. CMS-A18 and CMS-E18 differ only in the bond between core and inner shell (amide versus ester bond). Amide bonds are known to form hydrogen bonds and can induce highly ordered structures. This seems to be beneficial for a rigid and planar drug such as dexamethasone. Furthermore, hydrogen bonds can be formed between the core amide and hydroxyl groups of the guest molecule. These two factors resulted in an overall higher loading capacity for dexamethasone in CMS-A18 compared to CMS-E18. Due to its branching and structural diversity, CMS-E18b exhibited the least ordered hydrophobic segment, which also resulted in a low *T*_m_ (see Figure 2). Apparently, the low degree of order for the hydrophobic chains was not beneficial compared to the unbranched system CMS-E18 for encapsulation of the rigid drug dexamethasone.

**Figure 4:**
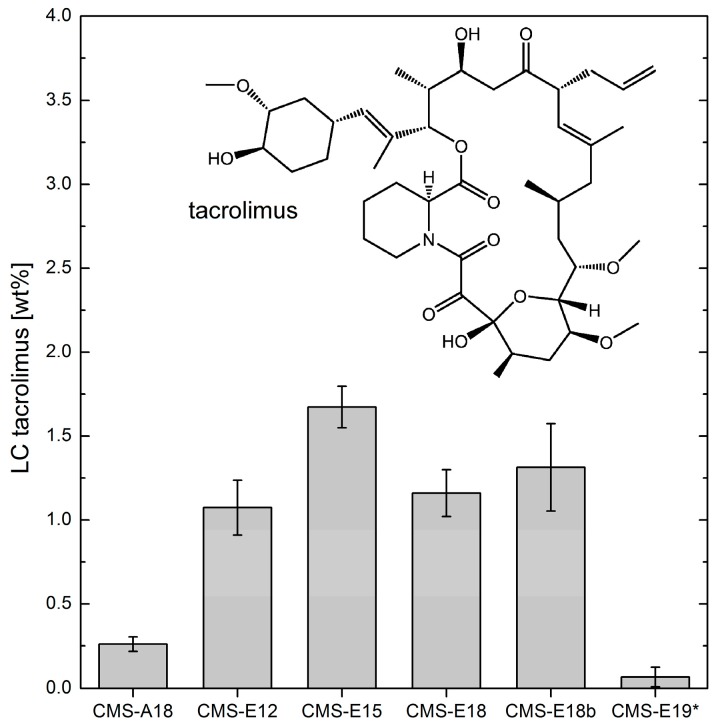
Loading capacities of tacrolimus in the examined carrier systems vs. the *T*_m_ values of the inner shell. Encapsulation was done with the film-uptake method, *n* = 3, error bars indicate standard deviation, * performed at 60 °C.

Employing the same method and a similar, HPLC-based analysis, the LCs for tacrolimus were determined as well (see Figure 4 and Table S4 for numerical values). Among the carrier architectures in discussion, there was a clear maximum at the inner shell length of 15 carbon atoms. This was due to two effects. Along the carriers, CMS-E12–CMS-E15–CMS-E18, the increasing hydrophobic interactions impeded higher drug loading such as in the case of dexamethasone. Additionally, the sterically demanding molecule tacrolimus required a minimum size of hydrophobic segments, which was too small in the case of CMS-E12. Hence, the optimum size in this row was 15 carbon atoms. 

Comparing CMS-A18 to CMS-E18, exchanging the ester bonds to amide bonds had a tremendous effect on the *LC*. The rather rigid segments of C18 chains in the amide-functionalized carrier impaired efficient encapsulation of the flexible and large macrocycle tacrolimus. This did not seem to be the case for CMS-E18. Three to fourfold higher *LC*s were measured for this system. CMS-E18b, which featured the least ordered hydrophobic segment, had a similar *LC* to CMS-E18. The branching of the inner shell and a longer PEG outer shell was not beneficial for the encapsulation of tacrolimus. 

### 3.6. Enzymatic Degradation of Unloaded Carriers

**Figure 5:**
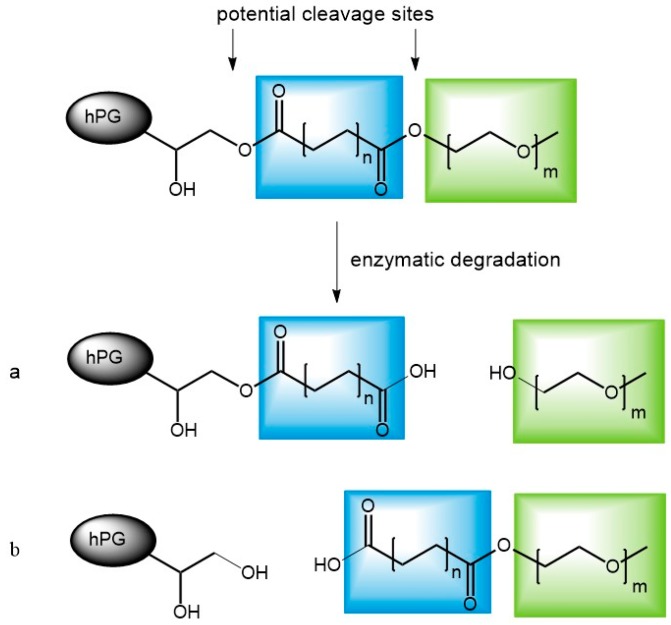
Different cases of ester cleavage. Depending on the site of cleavage, either the double shell (**a**) or the PEG-chain (**b**) is cleaved off.

**Figure 6:**
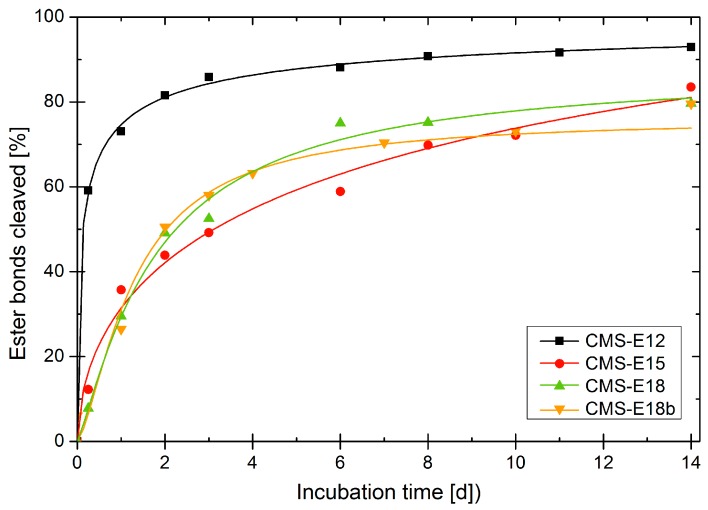
Comparison of the degree of hydrolysis for the ester bonds by lipase from Rhizomucor miehei as determined by NMR (DMSO-d_6_), *n* = 1.

The rate at which the carrier is degraded enzymatically was determined by adding a lipase to a solution of nanotransporter in PBS and taking samples at certain time points. The commercially available lipase from Rhizomucor miehei was chosen because of the resemblance of the inner shell building blocks to fatty acids, its native substrate. The samples were lyophilized, redissolved in DMSO-d_6_ and analyzed in ^1^H NMR. The α-protons and the methylene group adjacent to the ester group on the PEG side were tracked in the ^1^H NMR spectra (the protocol was adapted from a method published earlier by our group) [29]. The chemical shift of the α-protons depends on whether the carboxylic acid was esterified or not. At *t* = 0, there were only the peak at 2.23 ppm, which represented the 4 esterified α-protons, as well as the peak at 4.07 ppm, that originated from the two PEG protons adjacent to the ester bond. These signals indicated ester bonds only. Upon degradation, the peak at 4.07 ppm decreased and two new peaks appeared and started to grow over time in the NMR spectrum (see Figure S4). One was a triplet at 2.17 ppm and the other one was a broad signal at 2.12 ppm. Both represent non-esterified α-protons, the triplet originated from a non-carrier bound carboxylic functionality, while the broad signal can be attributed to a carboxylic function still bound to the nanocarrier architecture. Hence, ester cleavage is reflected in the signals of the α-protons. Whether the inter-shell ester or the core-ester is cleaved (Figure 5), can be determined by tracking the PEG protons at 4.07 ppm. By cleavage at the inter-shell site, this signal will lose intensity. The results of this analysis are plotted in Figure 6 and complement a previous study examining the degradation of the nanocarriers by skin lysate [22]. The graph shows degradation of more than 70% of all ester groups for all carriers after six days. Since the used method not only allows one to follow the total cleavage of all ester groups but also the individual monitoring of cleavage sites, one can see which bond was cleaved first (Figure 5 and Figure 6 and Figure S5). 

Surprisingly, the core ester bond was cleaved at a comparable rate as the inter-shell ester. In the first 24 h, almost no inter-shell esters cleaved. The reaction rate was very similar after that. The simultaneous cleavage of core and inter-shell ester showed that an enzyme of a molecular weight of 29.6 kDa still could enter the spherical particle and catalyze ester cleavage, which indicated the high flexibility of the CMS nanocarriers. In contrast, no cleavage could be observed for the amide-based CMS nanotransporters under the same conditions (see Figure S6). This was surprising because the CMS-A18 also has esters as the linkage of the diacid with the mPEG and hence ester cleavage should have been still possible to a certain degree.

### 3.7. Release Mediated by Enzymatic Cleavage

The guest molecule is encapsulated in the CMS nanocarrier by physical entrapment, which leads to its solubilization. Its release is realized by diffusion out of the nanoparticle into the surrounding medium. A second mechanism is the degradation of the carrier in a confined volume upon which it will lose its ability to solubilize hydrophobic drugs. In order to determine the kinetics of this second mechanism, the particles were loaded with dexamethasone and enzymatically degraded. The released drug precipitated, and was separated by centrifugation. The reduced drug content of the supernatant was measured by HPLC to determine the remaining solubilizing effect of the partially degraded carrier. After resuspension, the procedure was repeated at different time points. In this procedure, we focused on the best performing carrier systems, CMS-E12, CMS-E15 and CMS-E18. In order to only determine the surplus value of the CMS nanotransporter, from each value, c(sup) and $c_{t=0}\left(sup\right)$, the natural solubility of Dexamethasone, $c_{sat}\left(Dx\right)$, was deducted (Equation (4)). Then, the ratio of each value c(sup) to its initial value $c_{t=0}\left(sup\right)$ was calculated and subtracted from 1 to normalize the graphs. CR (cumulative release) represents the percentage of drug that was released from the carrier due to degradation.

(4) $$CR=1-\frac{c\left(sup\right)-c_{sat}\left(Dx\right)}{c_{t=0}\left(sup\right)-c_{sat}\left(Dx\right)}$$

**Figure 7:**
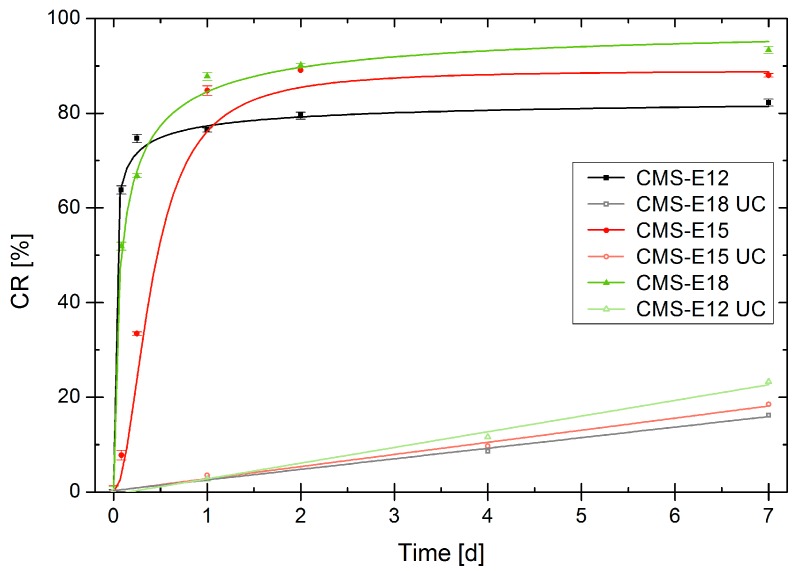
Increase of drug release upon degradation in comparison to the untreated control (UC). CMS nanocarrier concentration 10 mg·mL^-1^, *n* = 3.

This protocol was carried out with double carrier concentration to increase the effect but the carrier/enzyme ratio was kept identical to get a comparable time scale in which the degradation took place. Figure 7 indicates that all carriers underwent a rapid change in their transport properties. The solubilization enhancing properties strongly diminished within a degradation time of one day. In the same period, only around 3% of the payload was released in the UC and around 20% in seven days.

## 4. Discussion

By introducing the ester linkage between core and inner shell of CMS nanocarriers, an easier synthetic route was established. The synthesis was reduced to three synthetic steps as compared to six for the amide-based system. The resulting CMS nanocarriers showed different aggregation phenomena from its amide-functionalized counterpart. These properties were strongly linked to the loading capacities of the carriers. While a short inner shell of 12 carbon atoms and amide bonds were beneficial for encapsulation of the rigid molecule dexamethasone, the bulkier tacrolimus showed a clear optimum at an inner shell chain length of 15 carbon atoms which was attached via an ester linkage. Furthermore, ester-based carriers showed a high degree of enzymatic degradability. Hence, this new class of ester-based CMS nanocarrier presented here combines optimized loading capacities for the anti-inflammatory hydrophobic drugs dexamethasone and tacrolimus, easy synthesis, and enzymatic degradability.

## Figures and Tables

**Table 1:** Estimation of the degree of functionalization (NMR) and GPC analytical data.

Compound	*M*_n_ [kDa]	*PDI*	*DF* (NMR)
CMS-A18	41.8	1.85	65%
CMS-E12	32.2	1.31	88%
CMS-E15	43.1	1.79	90%
CMS-E18	34.9	1.41	88%
CMS-E19	32.3	1.31	75%
CMS-E18b	33.3	1.68	69%

**Table 2:** Intensity-based hydrodynamic diameters of unloaded and loaded CMS nanocarriers and the ζ potential of unloaded CMS nanocarriers. Samples had a concentration of 5 g·mL^−1^ polymer and were measured at 25 °C. Experiments were performed in triplicates.

Compound	Unloaded		Tacrolimus-Loaded		Dexamethasone-Loaded		ζ Potential
	*d* [nm]		*d* [nm]		*d* [nm]		[mV]
CMS-A18	15	(81%)	19	(5%)	32		0.07 ± 0.09
	210	(19%)	204	(95%)			
CMS-E12	14	(48%)	14	(5%)	63	(10%)	−1.27 ± 1.04
	134	(52%)	215	(95%)	361	(90%)	
CMS-E15	15	(23%)	16	(10%)	16	(9%)	0.01 ± 0.06
	138	(77%)	208	(90%)	270	(91%)	
CMS-E18	37		18	(15%)	24	(25%)	−5.9 ± 0.7
			224	(85%)	272	(74%)	
CMS-E19 *	216		364		47	(9%)	0.01 ± 0.03
					329	(91%)	
CMS-E18b	76		123		121		−0.003 ± 0.06

* Measured at 60 °C.

## Supplementary Materials

Supplementary materials are available online at http://www.mdpi.com/2073-4360/9/8/316/s1.

## Abbreviations

CMC	Critical micellar concentration
CMS	Core–multishell
CR	Cumulative release
DDS	Drug delivery system
DF	Degree of functionalization
hPG	Hyperbranched polyglycerol
hPG–NH_2_	Hyperbranched polyglycerol amine
ITCC	Indotriscarbocyanine
PEI	Poly(ethyleneimine)
