# Supplementary material for: Biodegradable Core–Multishell Nanocarriers: Influence of Inner Shell Structure on the Encapsulation Behavior of Dexamethasone and Tacrolimus
Source: Polymers (Basel). 2017 Jul 29;9(8):316. doi: 10.3390/polym9080316 (PMC6418772; doi:10.3390/polym9080316)
Supplement: Supplementary file 1 [file polymers-09-00316-s001.pdf]

Supporting Information

Biodegradable Core-Multishell Nanocarriers: Influence of Inner Shell Structure on the Encapsulation Behavior of Dexamethasone and Tacrolimus

Michael L. Unbehauen [1], Emanuel Fleige [1,2], Florian Paulus [1,2], Brigitta Schemmer [3], Stefan Mecking [3], Sam Dylan Moré [2] and Rainer Haag [1,*]

[1] Freie Universität Berlin, Institute for Chemistry and Biochemistry, Takustraße 3, 14195 Berlin, Germany
[2] DendroPharm GmbH, Arnimallee 14, 14195 Berlin, Germany
[3] Chemical Materials Science, Department of Chemistry, University of Konstanz, Universitätsstraße 10, 78467 Konstanz, Germany
* Correspondence: haag@chemie.fu-berlin.de; Tel.: +49-30-838-52633

1. **Material and Methods**

Compound	Fraction
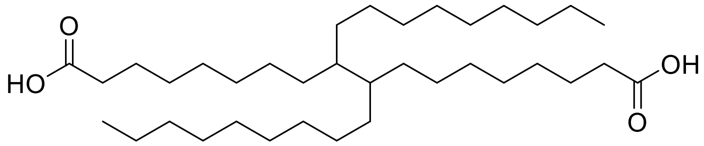	~30%
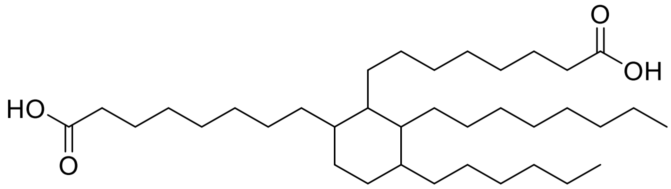	~50%
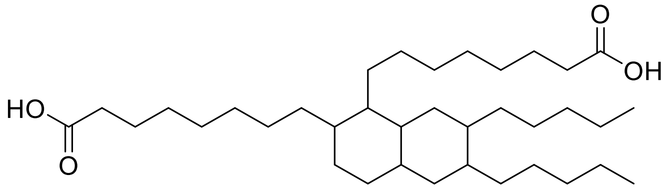	
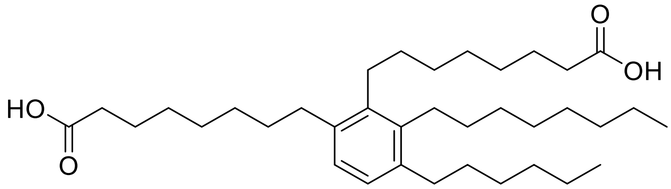	~20%
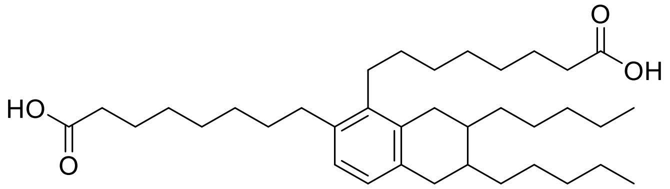	

Scheme S1. Constituents of the dimeric fraction of EMPOL 1026.

The compound C18b is a mixture of different isomers of the dimeric fraction of the product EMPOL 1026 from Cognis. The dimeric fraction was isolated by column chromatography with hexane/ethyl acetate on acidified (acetic acid) silica. The dimeric fraction comprises of the compounds that are given in Scheme S1 and its isomers.

2. Degree of Functionalization

Table S1. Estimation of the degree of functionalization from GPC analytical data based on M_w.

compound	DF (GPC)
CMS-A18	42%
CMS-E12	20%
CMS-E15	45%
CMS-E18	24%
CMS-E19	29%
CMS-E18b	42%

2.1. Calculation of DF via NMR

All signals between 4.48 ppm and 3.32 ppm and an additional peak at 5.20 ppm can be attributed to all methylene and methine protons of hPG (Figure S1, a) and is partially overlaid with peaks assigned to the PEG backbone. (Figure S1, e,f,g) The aliphatic signal at 1.34 ppm, which has 18 protons (d), is needed to estimate the fraction that is assigned to the hPG backbone (a).

Per glycerol unit, the signal (a) originates from 5 protons of the hPG backbone and, depending on the degree of functionalization (DF), another 28.8 protons are in position e + f at the PEG backbone and additional 3 at position g (Equation S1 and S2). The signal of the 18 protons in (d) also depends on the DF (Equation S3).

Having established these relations (S1-3), the focus is now on the single double-shell chain. Scaling the peak (d) ppm to 18, one can now calculate the relation between DF and $\sigma_{a,e,f,g}$ (Equation S4) using Equation S2 and S3. This equation is then simplified to Equation S5 and later solved for DF to yield Equation S6, which now can be used to calculate DF from $\sigma_{a,e,f,g}$ after having set σ_d to 18. This procedure works equally for CMS-A18, for CMS-E12 σ_d set to 12, for CMS-E18 to 24, and for CMS-E19 to 26. An adapted form of this equation (Equation S7) was used to calculate DF of CMS-E18b. In this case, the methoxy peak at 3.36 ppm was set at 3.

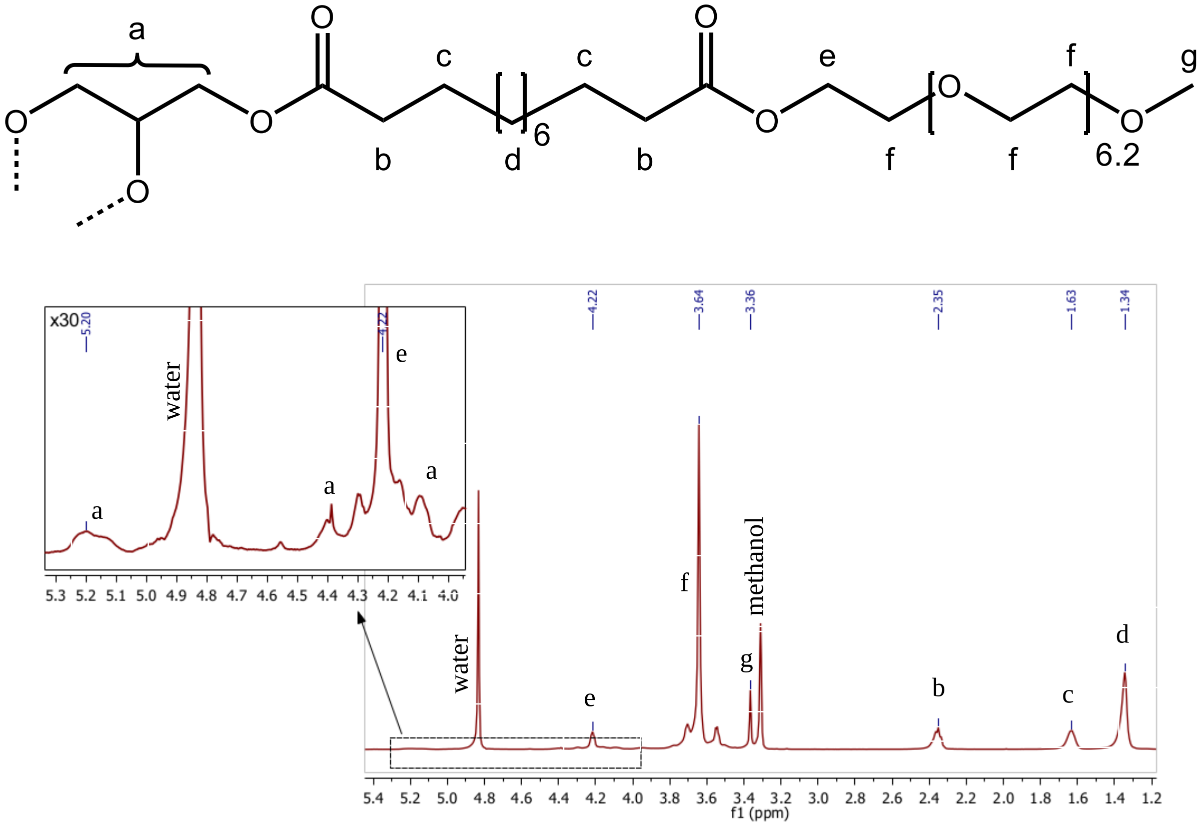

Figure S1. Exemplary chemical structure of a functionalized glycerol unit (top) and the respective ^{1}H-NMR spectrum (bottom) of CMS-E12.

$$\sigma_{a,e,f,g} = 5 + 7.2 \cdot 4 \cdot DF + 3 \cdot DF \tag{S1}$$

$$\sigma_{a,e,f,g} = 5 + 31.8 \cdot DF \tag{S2}$$

$$\sigma_d = 18 \cdot DF \tag{S3}$$

$$\sigma_{a,e,f,g} = \frac{\sigma_{a,e,f,g}}{\sigma_d} \cdot 18 = \frac{5 + 31.8 \cdot DF}{DF} \tag{S4}$$

$$\sigma_{a,e,f,g} = \frac{5}{DF} + 31.8 \tag{S5}$$

$$DF = \frac{5}{\sigma_{a,e,f,g} - 31.8} \tag{S6}$$

$$DF = \frac{5}{\sigma_g - 63.2} \tag{S7}$$

2.2. *Accuracy of the NMR experiment*

Assuming an average measurement error (ME) of 3 %, the deviation for the measured DF was calculated depending on the real DF (see Figure S2) using Equation S8. This Equation combines Eq. S5

and S6 and estimates the deviation by converting a theoretical DF into an NMR integral σ, adding or subtracting the measuring error (ME) and converting it back. Equation S8 is valid for CMS nanocarriers using mPEG350, S9 for CMS-E18b. As depicted in Fig. S2, the range of error increases with higher DF, leading to a rather rough estimation for highly functionalized CMS architectures.

$$DF_{min/max} = \frac{5}{\left(\frac{5}{DF_{real}} + 31.8\right) \cdot (1 \pm ME) - 31.8} \quad \text{(S8)}$$

$$DF_{min/max} = \frac{5}{\left(\frac{5}{DF_{real}} + 63.2\right) \cdot (1 \pm ME) - 63.2} \quad \text{(S9)}$$

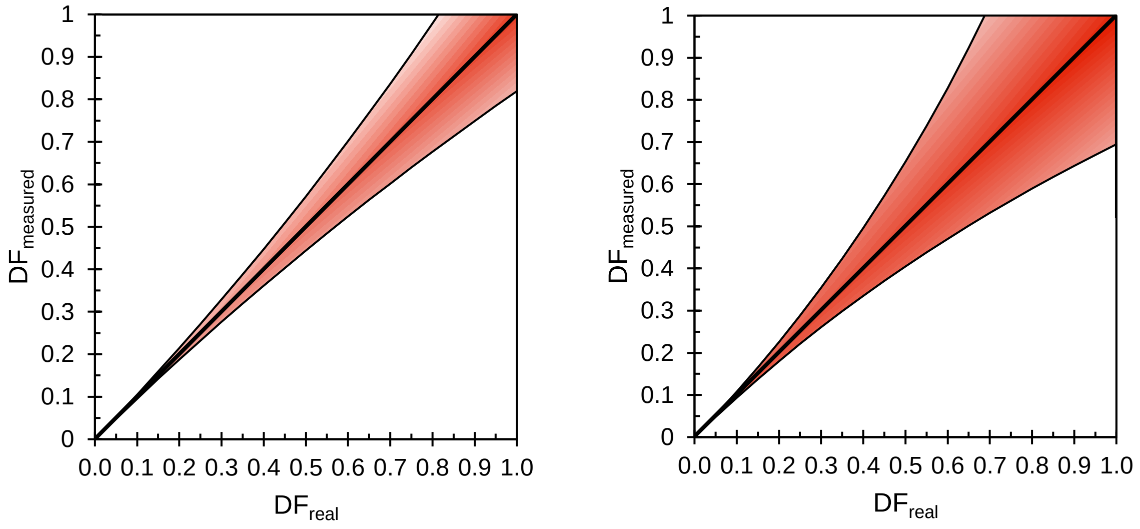

Figure S2. Estimated range of deviation (red) based on a measuring error of the NMR-experiment of 3% for CMS with mPEG350 (left) and mPEG750 (right).

3. Hydrodynamic sizes

Table S2. Hydrodynamic diameters of CMS in methanol determined by DLS.

Compound	Size (DLS, diameter, methanol, number)
CMS-A18	6.4
CMS-E12	8.5
CMS-E15	5.9
CMS-E18	8.6
CMS-E19	6.9
CMS-E18b	8.2

Table S3. Hydrodynamic diameters of CMS water, before and after filtration, and in PBS determined by DLS, percentage of signal in brackets.

Compound	Size before filtration	Size after filtration	Size after addition of PBS
CMS-E12	14.5 nm (43%)<br>228 nm (54%)	13.8 nm (48%)<br>143 nm (52%)	14.8 nm (48%)<br>143 nm (48%)
CMS-E15	14.1 nm (27%)<br>113 nm (73%)	16.3 nm (31%)<br>145 nm (69%)	20.6 nm (36%)<br>154 nm (64%)
CMS-E18	42.3 nm	39.2 nm	39.6 nm

The hydrodynamic diameter was measured before and after filtration with a 450 nm RC filter and after the dissolution of PBS salt. The sizes and populations widely remained the same, only in CMS-E12 the aggregates' diameters decreased.

4. Loading Capacity vs. T_m of the inner shell

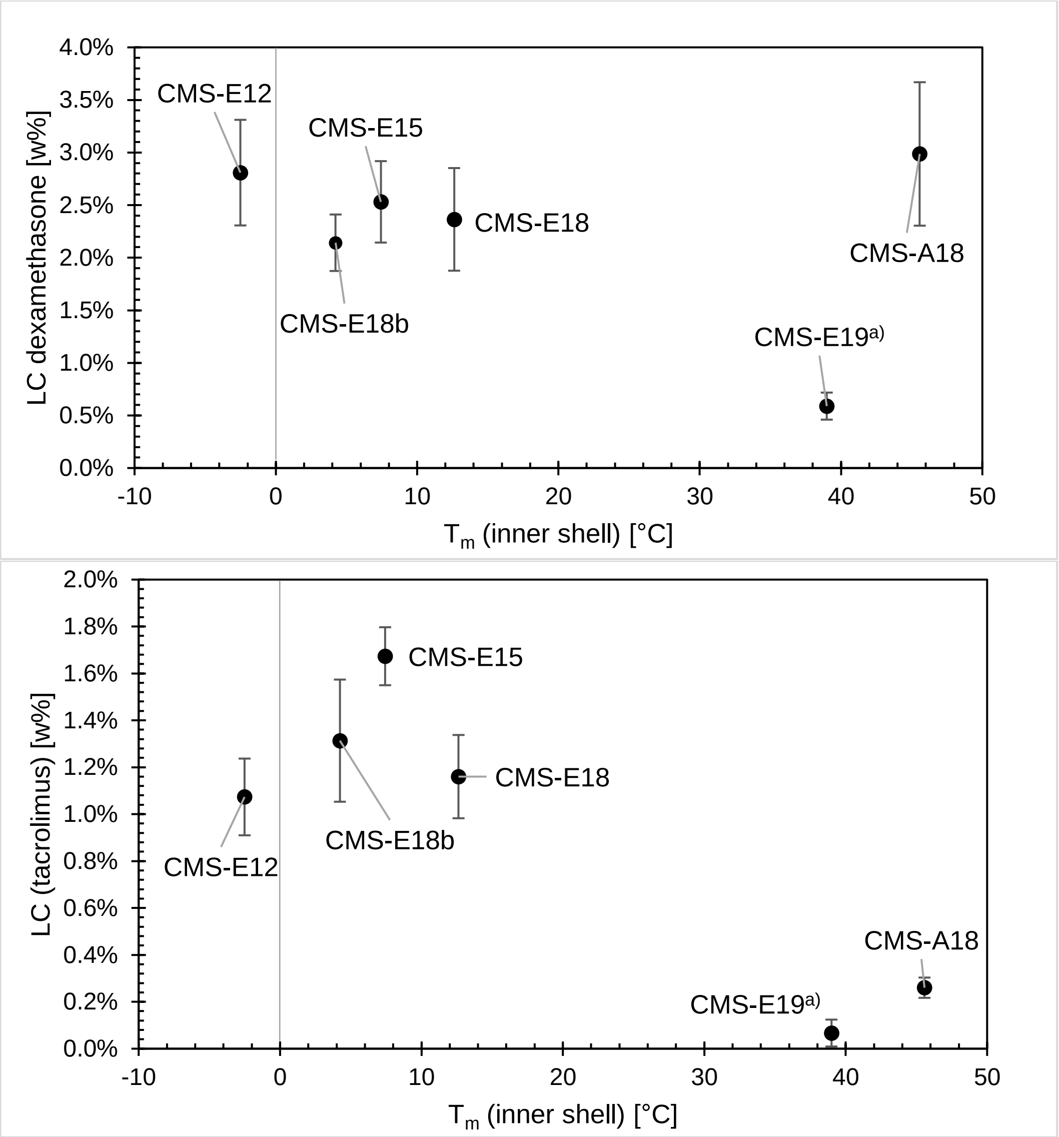

Figure S4. Loading capacities of the investigated carrier architectures for dexamethasone (top) and tacrolimus (bottom) plotted against the melting temperature of their respective inner shell. No correlation could be found. [a)]Encapsulation performed at 60 °C.

Table S4. Loading capacities of dexamethasone and tacrolimus.

compound	loading capacity<br>dexamethasone [wt%]	tacrolimus [wt%]
CMS-A18	3.0 ± 0.7	0.26 ± 0.1
CMS-E12	2.8 ± 0.5	1.1 ± 0.2
CMS-E15	2.5 ± 0.4	1.7 ± 0.1
CMS-E18	1.7 ± 0.5	1.16 ± 0.1
CMS-E19*	0.6 ± 0.1	0.07 ± 0.1
CMS-E18b	2.1 ± 0.3	1.3 ± 0.3

*measured at 60°C

5. Enzymatic degradation of CMS-E

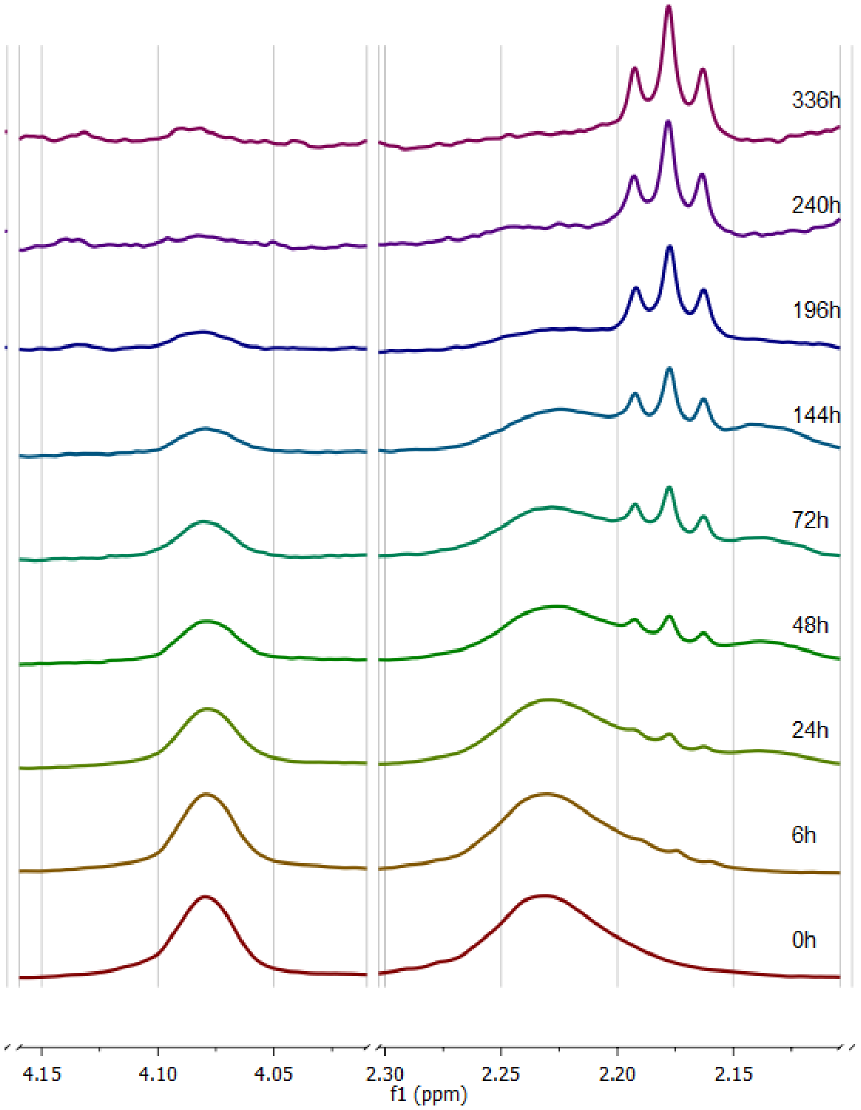

Figure S4. Relevant NMR signals for determining the rate of degradation in DMSO-d6: The signal at 4.07 ppm vanishes over time, indicating a cleavage of the inter-shell ester. The signal at 2.23 decreases

upon any ester cleavage and reappears either as a triplet at 2.18 ppm (cleavage of the inter-shell ester) or as a broad signal at 2.14 ppm (cleavage of core ester).

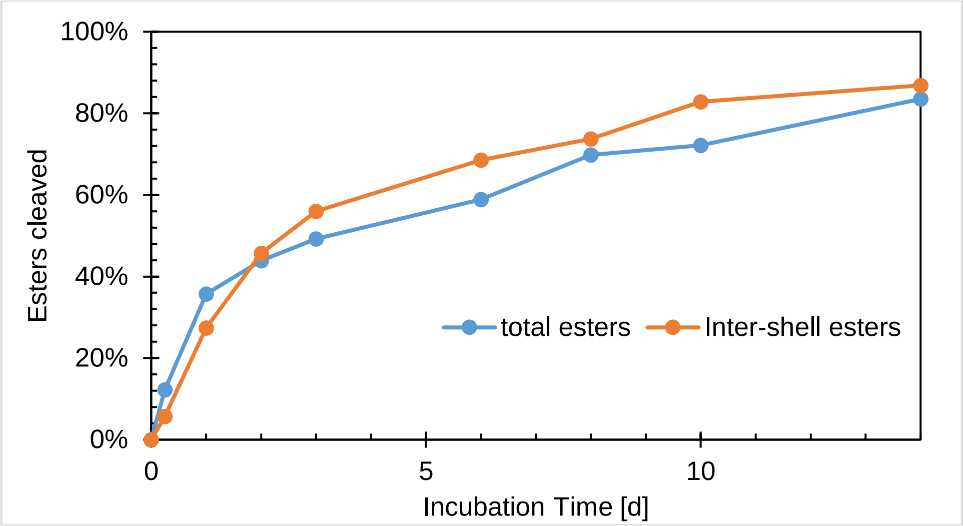

Figure S5. Degree of cleavage of total ester bonds (blue) and inter-shell ester bonds of CMS-E15 as determined by NMR (DMSO-d6).

6. Enzymatic degradation of CMS-A18

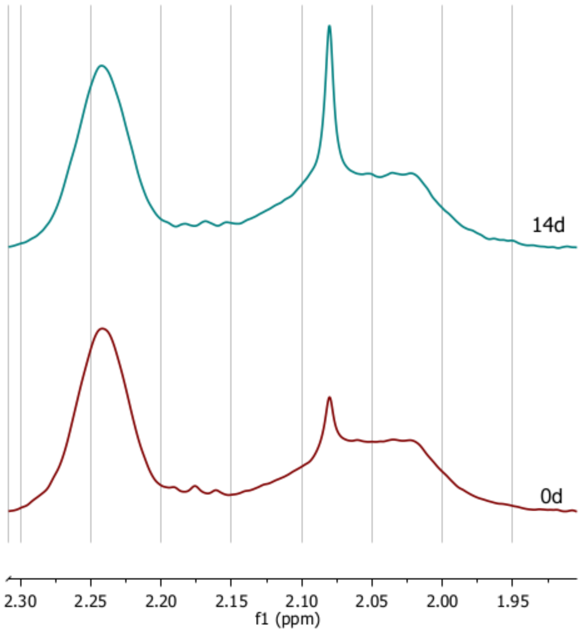

Figure S6. Comparison of the relevant peaks to determine the degree of degradation in DMSO-d6 at the start (bottom) and after 14 days (top): The peak at 2.24 ppm can be assigned to the ester α-protons and

the triplet at 2.18 ppm to the α-protons of the acid. The broad peak between 2.12 ppm and 1.97 ppm is assigned to the amide α-protons and is overlapped by an impurity at 2.08 ppm. There is no decrease of the amide or ester peaks over time in favor of the signal of the acid protons over time.

7. Release mediated by enzymatic cleavage

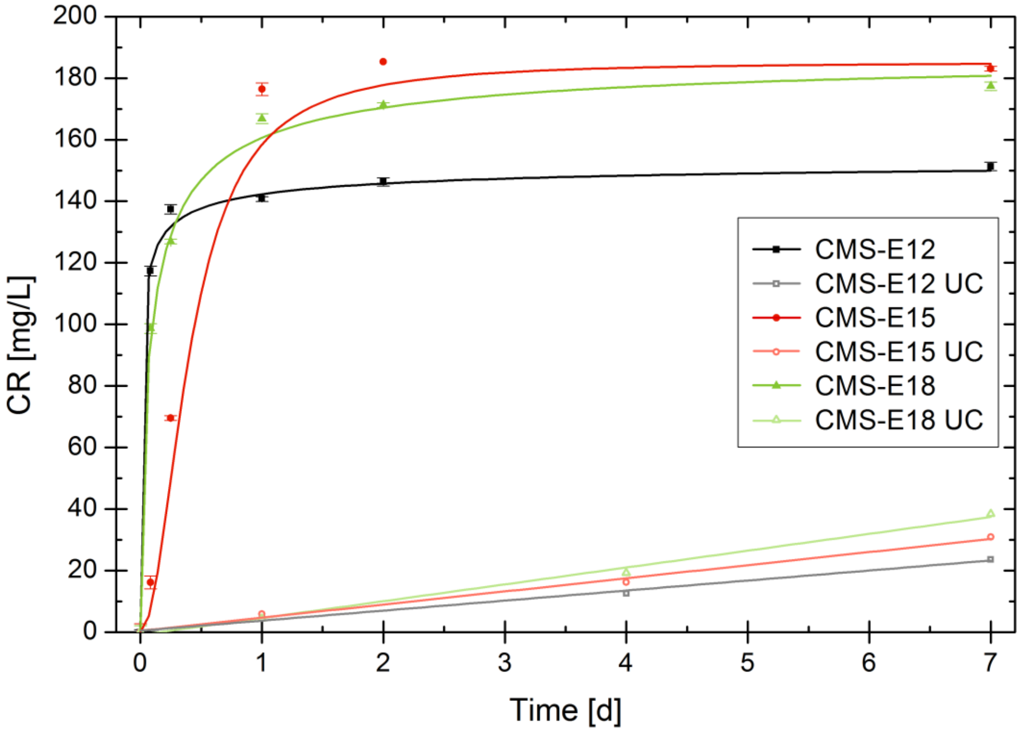

Figure S7. Cumulative Release (CR) of the absolute amounts of dexamethasone from the solutions of loaded carriers (polymer concentration 10 g/L) in comparison to the untreated control (UC) without enzyme, n=3.
